# Structural Response Prediction for Damage Identification Using Wavelet Spectra in Convolutional Neural Network

**DOI:** 10.3390/s21206795

**Published:** 2021-10-13

**Authors:** Edisson Alberto Moscoso Alcantara, Michelle Diana Bong, Taiki Saito

**Affiliations:** Department of Architecture and Civil Engineering, Toyohashi University of Technology, Aichi 441-8580, Japan; moscoso.alcantara.edisson.alberto.eh@tut.jp (E.A.M.A.); michelle.diana.bong.tg@tut.jp (M.D.B.)

**Keywords:** convolutional neural network, wavelet spectrum, damage identification, structural health monitoring, sparse accelerometers

## Abstract

If damage to a building caused by an earthquake is not detected immediately, the opportunity to decide on quick action, such as evacuating the building, is lost. For this reason, it is necessary to develop modern technologies that can quickly obtain the structural safety condition of buildings after an earthquake in order to resume economic and social activities and mitigate future damage by aftershocks. A methodology for the prediction of damage identification is proposed in this study. Using the wavelet spectrum of the absolute acceleration record measured by a single accelerometer located on the upper floor of a building as input data, a CNN model is trained to predict the damage information of the building. The maximum ductility factor, inter-story drift ratio, and maximum response acceleration of each floor are predicted as the damage information, and their accuracy is verified by comparing with the results of seismic response analysis using actual earthquakes. Finally, when an earthquake occurs, the proposed methodology enables immediate action by revealing the damage status of the building from the accelerometer observation records.

## 1. Introduction

Earthquakes in the proximity of structurally vulnerable buildings could cause damage of varying intensities. Damage of different risk levels is often difficult to classify rapidly, making it difficult to accurately determine the structural safety of a building. For example, according to the National Institute of Civil Defense of Peru, during the Pisco earthquake on 15 August 2007, in the five main regions of Peru (Lima is included, which is the capital of Peru), 136,149 dwellings, 1278 educational buildings, and 126 health buildings collapsed or were damaged, and their use was classified as restricted or unsafe [1]. However, this report was released almost two months after the earthquake, during which time, all activities in the affected areas of the main regions had to be suspended, including the construction of temporary dwellings.

Resilient cities are goals that countries are building towards to increase the capacity for learning from past disasters for better future protection and to improve risk reduction measures [2]. In particular, as part of this concept, there is a need to develop modern structures for which we can quickly obtain the structural safety information after an earthquake for resuming economic and social activities in order to minimize social disruption and mitigate the effects of future earthquakes [3]. In order to promote and disseminate knowledge to increase social resilience and reduce earthquake risk, experts from academia and industry gathered in 2019 for a workshop focused on state-of-the-art risk-reduction strategies. It identified a need for research in the area of structural health monitoring (SHM) to assess the integrity and performance of engineering structures in order to quickly detect damage after an earthquake and enable decision making [4]. SHM is a field where it is possible to obtain the real-time structural responses and successful fast post-earthquake damage detection of monitored buildings, bridges, cultural heritage structures, dams, base-isolated buildings, etc. [5,6]. For instance, Goulet et al. proposed a methodology that updates the prediction of the damage state of uninspected monitored buildings as the model learns from collected data of the damage state of inspected buildings [7]. This proposal was validated in a city with 1000 buildings. Furthermore, Sivasuriyan et al. reviewed a large number of studies on the practical implementation and operations of SHM in multi-story buildings, as well as damage evaluation of monitored buildings, and discussed the structural response by considering static and dynamic analysis using numerical simulations such as finite element analysis (FEA) [8].

In the field of SHM, there are several types of sensors to measure and diagnose the static and dynamic properties of the monitored buildings. Antunez et al. demonstrated that optical fiber sensors can be useful in the static and dynamic monitoring of large raw earth masonry structures common in cultural, historical, and architecturally recognized buildings around the world [9]. Piezoelectric sensors are another type of monitoring device, and Roghaei et al. proposed a method to identify stress and deformation using an array of sensors mounted in certain locations [10]. They verified the proposed method using a three-story steel building and confirmed that continuous monitoring and analysis of sensor signals can help the building manager to apply warning alarms and call for evacuation. However, the most common monitoring control sensor is the accelerometer. For instance, Wang et al. developed a method to evaluate the story damage index (SDI) based on the modal frequency and mode shape obtained from the records of earthquake response of a building [11]. Furthermore, an approximate story damage index (ASDI) was developed without considering the information of the floor mass to identify the extent of damage to the story. Although it was possible to verify the damage index by some numerical simulations and the experimental data analysis established previously, it was necessary to calculate the modal frequency and mode shapes from the post-earthquake structural responses of each story and to compare with the values of the building before the earthquake. It is worth pointing out that a large number of sensors will require a high investment. For this reason, Xu et al. estimated the maximum drift and time histories of relative displacement in all stories of multi-degree-of-freedom (MDOF) structures considering only one accelerometer, verifying the effectiveness of the method by taking into account the robustness, installation location, and truncation error [12].

The machine learning method, which predicts the structural responses using a learning model specific to the structure, may provide higher accuracy by updating the model after each earthquake. According to study [13], there are two approaches for damage identification: model-driven methods and data-driven methods. In a model-driven approach, usually, a high-fidelity physical model of the structure is used to establish a comparison metric between the model and the measured data from the real structure to distinguish the damage condition from the normal condition. In a data-driven approach, a structural model is used as a statistical representation of the system, and the main algorithms developed for this purpose are those in the field of pattern recognition or, more broadly, machine learning. A convolutional neural network (CNN) is a tool for solving the problem of pattern recognition related to image and video recognition, classification, natural language processing, and others. Oh et al. studied a method of predicting the time histories of displacement of building structures from the measured acceleration responses on each floor based on a CNN, considering that the time series of acceleration structural response is similar to pixel-based image data (every acceleration value corresponds to one pixel), which is the basic input data in CNN [14]. The validation of their proposed method was from a numerical process using the ASCE benchmark model and an experimental test on a reinforced concrete (RC) frame structure. However, the structural model and dynamic responses used in the studies exhibited linear behavior. Tsuchimoto et al. proposed a rapid safety evaluation of multi-story buildings using sparse acceleration measurements [15]. Their proposed method predicts the maximum story drift ratio, and ultimately classifies the damage into three classes, namely “Safe”, “Restricted Use”, and “Unsafe” from a damage-sensitive feature (comparison between linear and nonlinear acceleration measurement responses) and ground acceleration as input data. Subsequently, Tsuchimoto et al. modified the previous method for high-rise buildings and validated considering an experimental test of a large-scale structure (1/3-scale 18-story steel building tested on the shaking table at E-Defense in Japan) [16].

There are two main characteristics observed on the ground motion records due to earthquakes. The first is the non-stationary characteristics in which the intensity of the ground motion varies with time; they are represented by the acceleration, velocity, and displacement. The second is the non-stationary characteristics in which the frequency content of the ground motion varies with time; they depend on several parameters such as magnitude, source and path effects, local site conditions, etc. [17]. Time–frequency distribution analysis is a method of obtaining a two-dimensional spectral function (there are several types of functions according to resources and needs) from a one-dimensional signal (ground motion or time–history structural response) that reflects the time and frequency of the original signal and is suitable to analyze the changes in the linear and nonlinear structural responses with only one function. For instance, Tao et al. used the matching pursuit decomposition algorithm to analyze the time–frequency distribution of the ground motion and verify the effect on the dynamic response of a nonlinear structure, and finally, this method reveals the effect of the ground motion on the nonlinear structural response [18]. Moreover, Cao et al. demonstrated the effect of energy concentration on the structural nonlinear response by using the wavelet transform to obtain a local spectrum and change the energy distribution over time for several earthquake records [19]. Spanos et al. analyzed the undamaged and damaged condition of a 20-story steel frame building using the harmonic wavelet transform applied to structural responses to obtain the variation of the effective natural frequencies due to the influence of the nonlinearity developed during the seismic event [20]. Balafas and Kiremidjian used the continuous wavelet transform of the input and output acceleration measurements to extract damage sensitive features for seismic damage estimation in civil structures [21]. Noh et al. proposed an extraction method of three damage-sensitive features using wavelet transform spectrum for structural damage diagnosis and applied them to experimental data of a reinforced concrete bridge column and a four-story steel moment-resisting frame structure [22]. In general, time–frequency distributions are two-dimensional spectral functions that can be used as input data for a CNN to predict dynamic issues related to structural engineering. For example, Xu et al. proposed a methodology to recognize and classify different types of vibrational events (digging, walking, vehicles passing, and damaging) [23]. First, they denoise the unknown signal and use the short-time Fourier transform (STFT) to obtain the time–frequency spectra and input them to the CNN for automatic feature extraction and classification. The proposed method used the support vector machine method to compare the obtained recognition rates of vibration events over 90% with the previous soft-max classifier. Dokht et al. used a CNN and STFT to consider a dataset of over 4900 earthquakes recorded over 3 years in Canada to classify between earthquake and noise signals. They also used another CNN and wavelet spectrum to classify and separate P from S waves and estimate their approximate arrival times [24]. Their results achieved an average accuracy of nearly 99% for both networks. Mousavi et al. proposed a detector based on a deep neural network (CNN belong to this field) called CNN-RNN Earthquake Detector (CRED), which is a network that combines a CNN and a recurrent neural network (RNN), specifically the bidirectional long-short-term-memory (LSTM) method, to learn the time-frequency characteristics of the dominant phases in an earthquake signal from three-component data recorded at a single station, having an accuracy of 99.95% [25]. In addition, Liao et al. proposed an identification method for a structural seismic response using a wavelet spectrum as input data in a CNN to distinguish the responses during an earthquake event under serviceability conditions [26]. Linear and nonlinear behaviors are considered in the research. According to previous studies, the CNN method in the SHM field has advantages over other methods in terms of higher accuracy by updating the model after each earthquake, flexibility to combine different methodologies, wide application areas, etc., however, it requires a large database of known data to train the model.

Previous studies have not fully investigated how to define the damage level of each floor of a structure from the time–frequency distribution of the observation data of a single sensor. The Japan Structural Consultants Association (JSCA), an organization of building structural engineers in Japan, uses three parameters of safety criteria used on the assessment of a building: absolute acceleration, ductility ratio, and story drift ratio [27]. Acceleration is related to damage in nonstructural components, and ductility and story drift ratio are related to damage in structural components. It is worth pointing out that the use of only one sensor implies a low-cost investment. This study proposes a methodology to predict the absolute acceleration, ductility ratio, and story drift ratio on each floor under earthquake conditions using machine learning. In the beginning, the earthquake responses of a model building are calculated under the scaled earthquake records with several intensities (scale factors). The level of intensity is established to obtain a range of linear and nonlinear behavior of the building. Then, wavelet spectra are developed from the structural response accelerations on the upper floor of the building. The wavelet spectra are the input data of a CNN model to predict the absolute acceleration, ductility ratio, and story drift ratio on each floor, which correspond to the damage of the nonstructural and structural components of the building.

This paper contains sections as follows: In Section 2, the basis and methods of the structural response prediction for damage identification are described, including the structural model of the case study, wavelet spectrum, convolutional neural network, input ground motion, and scale factor of records. Next, the application of the methodology is carried out by two processes: training and validation. The results and the comparison of the prediction and reference values of the case study are shown in Section 3. In Section 4, a summary and discussion of the research results are presented.

## 2. Structural Response Prediction Method

### 2.1. Structural Model and Structural Responses for Damage Identification

**Figure 1:**
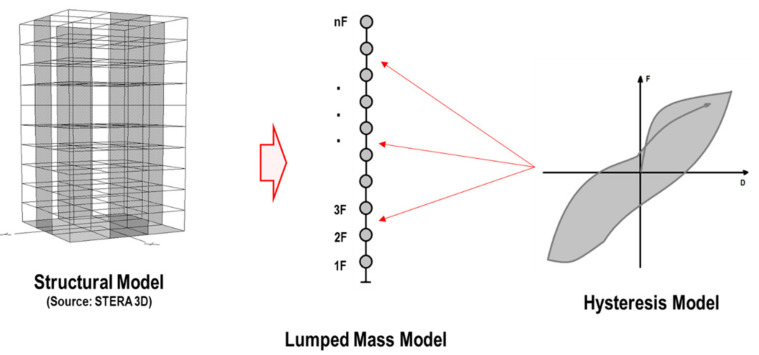
Structural model.

In this study, a lumped mass model (LMM) is considered as the structural model of the building, which takes into account the concentrated mass and the hysteresis model in each story of a low- to mid-rise building as shown in Figure 1.

**Figure 2:**
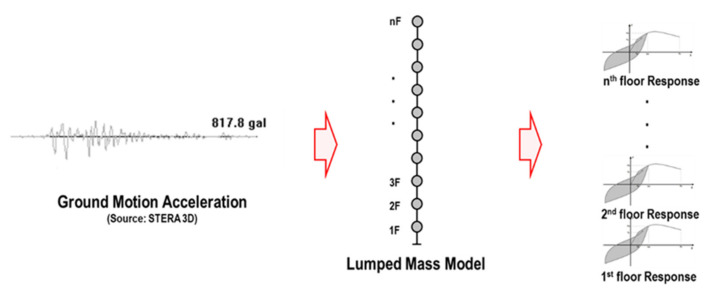
The general process of structural analysis.

The structural responses (displacement, acceleration, etc.) of each story of the LMM under the ground motion acceleration are obtained by a time history response analysis using the STERA_3D software [28]. The process is shown in Figure 2.

**Figure 3:**
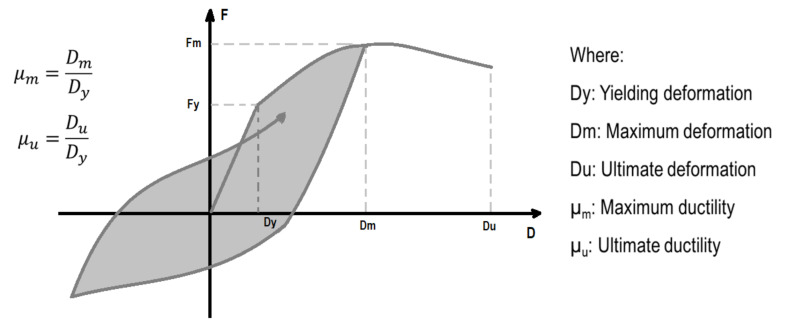
Definition of ductility ratio.

The maximum ductility ratio (ductility ratio from now on) indicates the amount of inelastic deformation over the yielding threshold as defined in Figure 3. This parameter is related to damage in the structural components of a building. Damage identification based on the ductility ratio is based on the performance-based guideline developed by JSCA [27] as follows: a ductility ratio <1.0 means no damage, a ductility ratio ≥1.0 but <2.0 means minor damage, a ductility ratio ≥2.0 but <3.0 means significant damage, a ductility ratio ≥3.0 but <4.0 means severe damage, and a ductility ratio ≥4.0 means collapse. These values are shown in Table 1. Notice that the ductility ratio is always greater than 1, however, in this study, ratios less than 1 are obtained as well to differentiate between the elastic and inelastic behavior.

**Figure 4:**
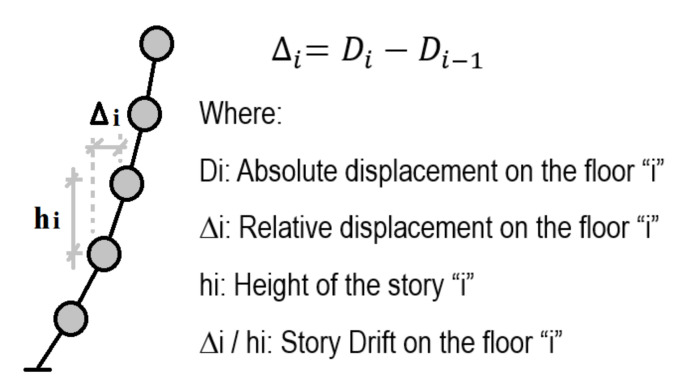
Definition of story drift ratio.

The maximum story drift ratio (story drift ratio from now on) represents the maximum relative displacement that a certain story reaches that is associated with the damage of structural components as defined in Figure 4. A larger story drift (relative displacement) after the yielding stage corresponds to a larger extent of damage. Damage identification based on the story drift ratio is based on the performance-based guideline developed by JSCA [27], as follows: a story drift ratio <1/300 means no damage, a story drift ratio ≥1/300 but <1/150 means minor damage, a story drift ratio ≥1/150 but <1/100 means significant damage, a story drift ratio ≥1/100 but <1/75 is severe damage, and a story drift ratio ≥1/75 means collapse. These values are shown in Table 1.

The maximum absolute acceleration (acceleration from now on) indicates the intensity that a certain story is subjected to and is associated with the damage of nonstructural components. Damage identification based on the acceleration is based on the performance-based guideline developed by JSCA [27], as follows: an acceleration <250 gal means no damage, acceleration ≥250 gal but <500 gal means minor damage, acceleration ≥500 gal but <1000 gal means significant damage, an acceleration ≥1000 gal but <1500 gal means severe damage, and an acceleration ≥1500 gal means collapse. These values are shown in Table 1.

Note that the damage condition after severe damage in all cases is considered collapse condition. Besides, no damage and minor damage represent a building that is safe for use, significant damage represents a building that can have restricted use, and severe damage represents a building that is unsafe for use, that is, a value greater than minor damage is a restricted or unsafe condition, which is a parameter used for evacuating the building.

### 2.2. Wavelet Spectrum

**Figure 5:**
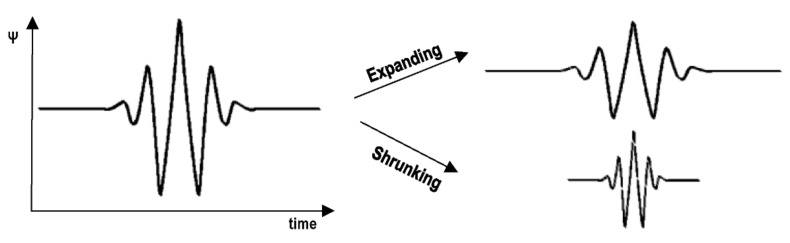
Types of dilation of the mother wavelet function.

**Figure 6:**
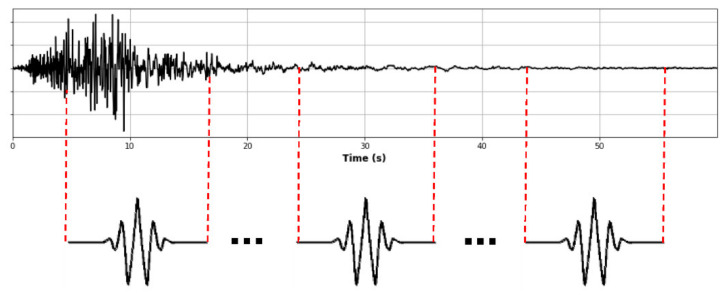
Translation of wavelets over time.

Various transformation functions are used to extract the characteristics of a signal. For example, the Fourier transform can be used to obtain the frequency components of a signal, but it cannot capture the changes over time. On the other hand, if the frequency component varies with time, there are methods such as using the instantaneous frequency or the short-time Fourier transform, both of which have the property that the resolution of time and frequency is constant. However, in actual analysis, it is often the case that low frequency components change slowly over time, while high frequency components change rapidly over time. In the wavelet transform, the optimal time and frequency resolution for each component can be obtained by changing the time resolution according to the frequency of the signal component (see Figure 5 and Figure 6, respectively).

**Figure 7:**
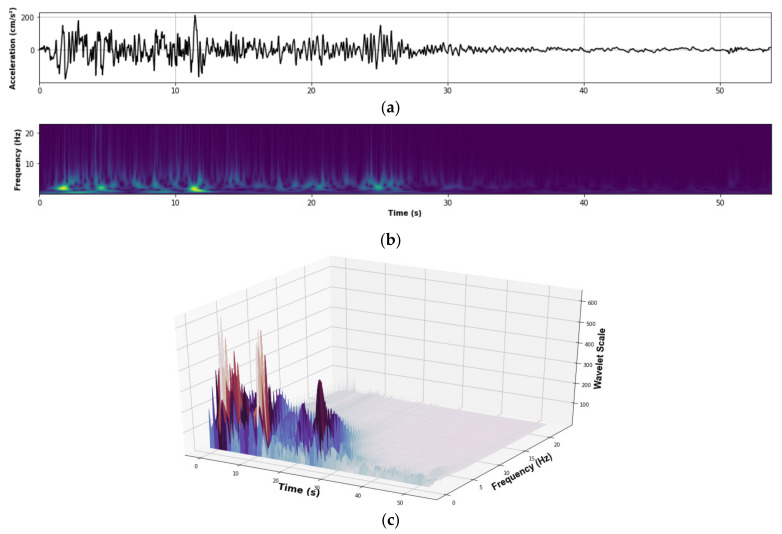
(**a**) Acceleration wave; (**b**) 2D wavelet spectrum; (**c**) 3D wavelet spectrum.

The continuous wavelet transform (CWT) of a signal *s*(*t*) is given by Equation (1):

(1) $$w\left(a,\;b\right)=\frac{1}{\sqrt{a}}\int_{-\infty }^{\infty }s\left(t\right)\psi^{*}\left(\frac{t-b}{a}\right)dt$$

where the function $\psi \left(t\right)$ is the mother wavelet (Morlet wavelet [29] used in this study), and “*a*” and “*b*” are dilation (scale) and translation (position) parameters, respectively [20]. The symbol (*) denotes complex conjugation. Therefore, the wavelet transform permits transformation from a signal to a spectrum (wavelet spectrum) in two dimensions (time and frequency) with coefficients (scales) that represent the intensity of the signal, in the time-domain and frequency-domain. The wavelet spectrum shows the highest intensity of the wave on the time-domain and frequency-domain only in one graph (Figure 7b). As a reference, Figure 7a shows the acceleration wave, and Figure 7c shows a 3D graph of the wavelet spectrum.

This is a powerful tool for extracting the characteristics of the waveform signals such as response acceleration, velocity, and displacement. Thus, the wavelet spectrum of the acceleration response waveform obtained from the accelerometer installed in the building is computed in this study and used as an input to the CNN model.

### 2.3. Convolutional Neural Network (CNN)

**Figure 8:**
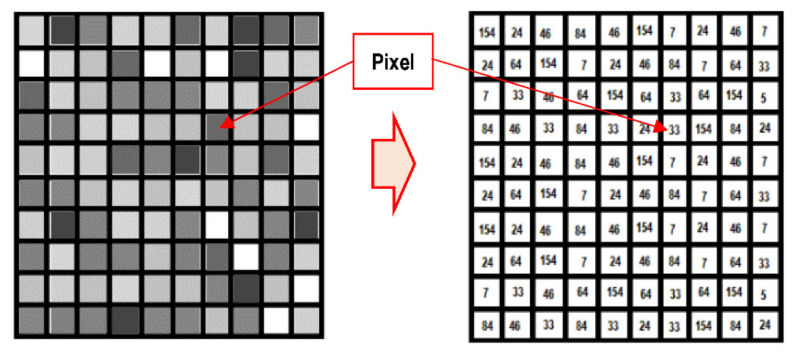
Digital image by an arrangement of pixels represented as numbers.

An image is processed by a computer as a grayscale image (image from now on) represented by an arrangement of numbers. For example, in Figure 8, the right matrix contains numbers between 0 and 255, each of which corresponds to the pixel brightness in the left image [30].

**Figure 9:**
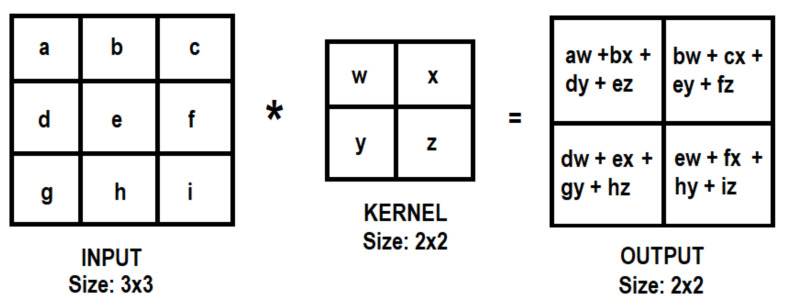
Convolution process of a part of an image by matrix multiplication. The symbol (*) means the convolution operator.

The convolution of the input image is performed by applying a set of weights, also known as a kernel or filter, as shown in Figure 9 [31].

**Figure 10:**
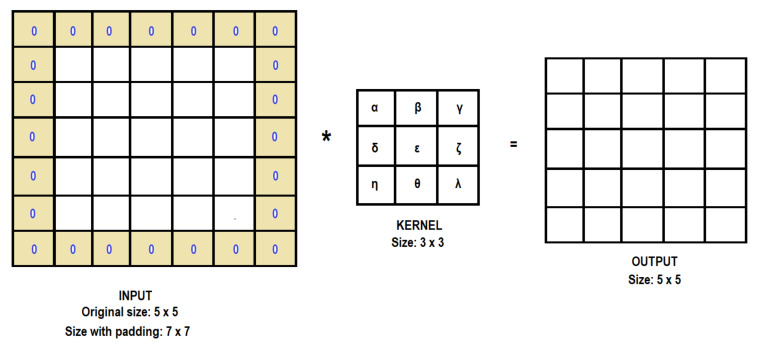
Padding same. The input image is filled with zeros along its border so that the output size is the same as the original input size. The symbol (*) means the convolution operator.

In the CNN method, images are used as input data, and for every input data set, the features of the input data are extracted by the convolution of the kernels. However, this convolution step loses information that might exist on the border of the image because they are only captured when the kernel slides (the kernel has to start and finish its process on the image borders) [32]. For this reason, the size of the input image is reduced as shown in Figure 9 (from input size: 3 × 3 to output size: 2 × 2). In order to obtain the same size as the original input, it is possible to apply the “same padding”, also called “zero-padding”, method (used in this study), which means the input is filled with zeros along its border as shown in Figure 10.

**Figure 11:**
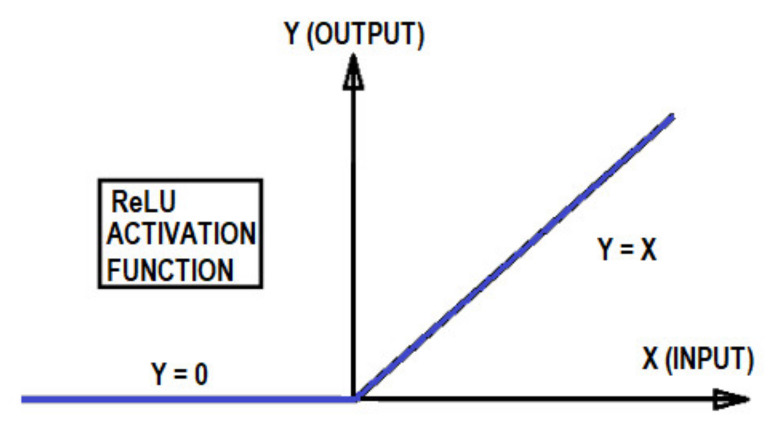
ReLU activation function.

Then, every resultant matrix is evaluated by a nonlinear activation function to allow for the learning of more complex models. The nonlinear activation function (activation function from now on) used in this study is the rectified linear unit (ReLU), defined as the function Y = max(X, 0) [33], as shown in Figure 11.

**Figure 12:**
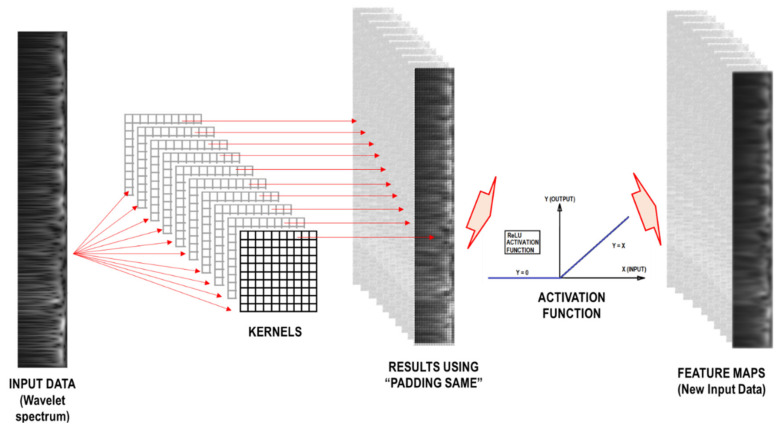
Typical convolutional layer, note that the input data is a representation of the wavelet coefficient’s matrix.

Finally, the new input data, the feature maps, are obtained. The process from the input data to the feature maps using the previous definitions is called the typical convolutional layer (see Figure 12).

**Figure 13:**
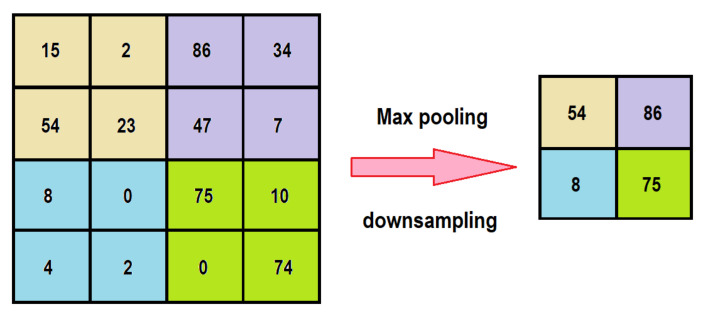
Maximum pooling process.

Usually, the typical convolutional layer is followed by a pooling layer to reduce the number of operations since the number of parameters increases as the network processes more kernels. A type of pooling layer is the “maximum pooling” or “max pooling” process, which takes the maximum value sliding along the feature map [30], as shown in Figure 13.

The pooling layer is required for image classification. It adjusts the features’ robustness to noise and disorder by reducing the resolution of the previous feature maps [33]. However, in this study, the CNN models with and without the maximum pooling layer were trained, and the CNN model without the maximum pooling layer converged on the output prediction more effectively. Therefore, the pooling layer is not used in the proposed CNN model.

**Figure 14:**
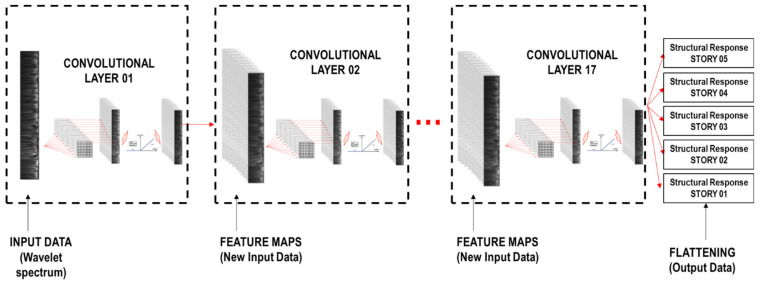
Convolutional neural network scheme. Structural response: ductility ratio, story drift ratio, or acceleration.

Usually, a hierarchical architecture is used in advance to propose the number of convolutional layers for the CNN architecture model [34]. In this study, after training the CNN models with different numbers of convolutional layers, 17 convolutional layers are finally used in the proposed CNN model, as shown in Figure 14.

Subsequently, the last convolutional layer is fully connected to the 1D layer or the flattening layer [33] (matrix of one column) with the number of stories as shown in Figure 14. In order to optimize the convergence and measure the error between the predicted and reference output, “Adam” [35] and mean squared error (MSE) are used as the optimizer function and the loss function. Equation (2) defines MSE, where *y_pred_* is the prediction output, *y_ref_* is the reference output, and *N* is the number of samples.

(2) $$\mathrm{MSE}=\frac{1}{N}\cdot \sum_{i=1}^{N}{\left(y_{pred}-y_{ref}\right)}^{2},$$

The flattening matrix contains structural responses for the damage identification, which can be the ductility ratio, story drift ratio, and acceleration (see Section 2.1 for their definitions). Figure 14 shows the CNN scheme used in this research.

Table 2 shows the architecture of the CNN for the structural response prediction method. This was finalized by extensive analysis of trained CNNs in advance. In Table 2, “No. kernels” is the number of filters or kernels assigned in each layer. Ten different kernels are used for the first layer and eight kernels are used for the other layers. Two types of kernel initializer are used in this study. “He_Normal” is used for the first four convolutional layers and “glorot_uniform” is used for the rest of the others. The kernel size is 10 × 10 for the first convolutional layer and 3 × 3 for the rest. The “same padding” and ReLU activation function are used in all convolutional layers.

**Figure 15:**
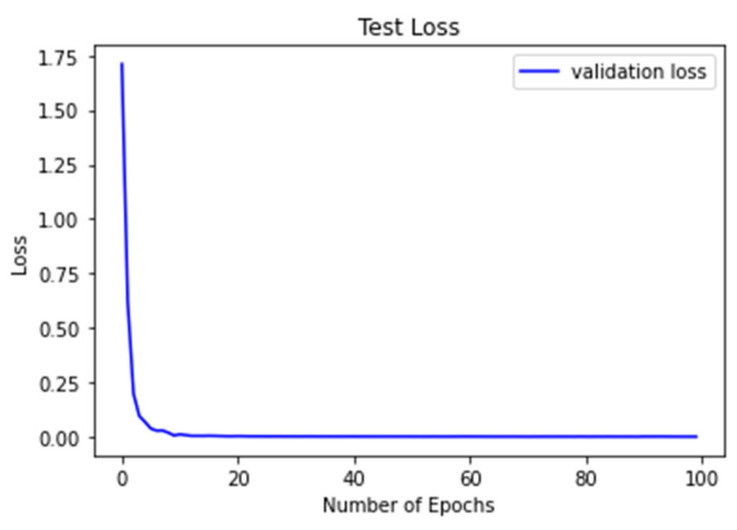
Converge curve of the trained CNN.

Figure 15 shows the convergence curve of the CNN model using the CNN architecture shown in Table 2, where “Loss” is the value of the loss function, and “Number of epochs” is the number of training iterations over the input data [31].

Firstly, the CNN model is trained with known input and output data. This is called the “training process”. Subsequently, new unknown input data are used to validate the trained CNN model by comparing the output data (structural responses for damage identification) with the reference structural responses. This process is called the “validation process” and the MSE function is used to evaluate the error.

### 2.4. Case Study and Input Ground Motion

#### 2.4.1. Case Study

The case study is a building of five stories with the following considerations (see Table 3 for more details):

- The fundamental period is considered the following: T_1_ = 0.025 H (H: total height of the building). The height of each story (h) is considered to be 4.0 m, then, H is 20 m and T_1_ is 0.5 s.
- LMM is used for the model of the building, and the bilinear hysteresis model (see Figure 5) is used to represent the nonlinear relationship between shear force and story drift for each story.
- The structural responses for damage identification (ductility ratio, story drift ratio, and acceleration) under earthquake ground motions are calculated by STERA 3D software [28].

**Figure 16:**
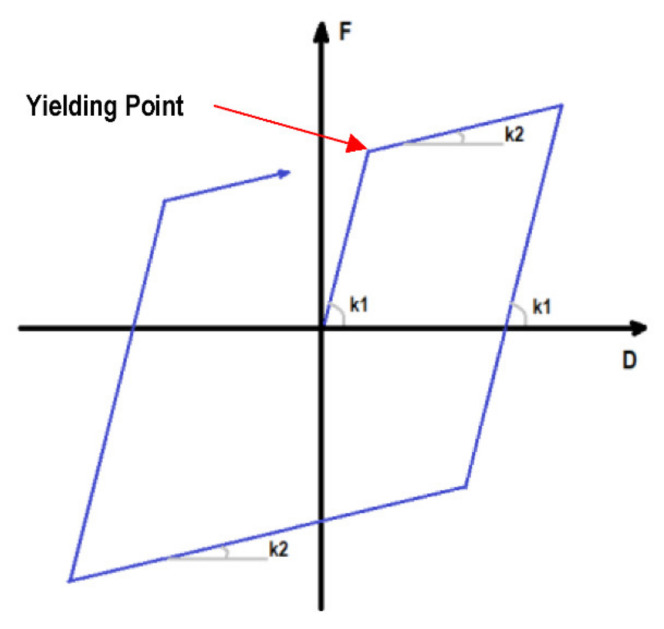
Bilinear hysteresis model for each story.

In order to build the bilinear hysteresis model, the yielding shear force (Qi) is calculated to be equal to the design shear force under the horizontal seismic load according to Japanese code. Moreover, the story stiffness (ki) is calculated so that the first mode shape becomes a triangular shape. Table 4 shows the parameters used in this study to define the bilinear hysteresis model in each story. The post-yield stiffness ratio (k2/k1, see Figure 16) is 0.1 for each story.

#### 2.4.2. Input Ground Motion

**Figure 17:**
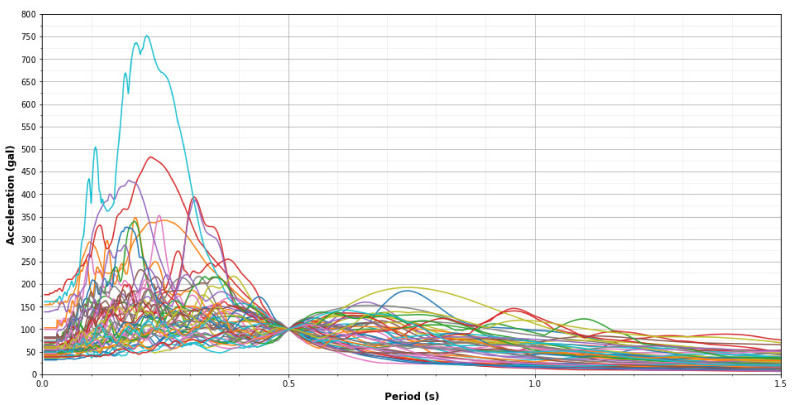
Acceleration response spectrum of 50 records scaled to have the same values at the fundamental period T_1_ = 0.5 s.

Table 5 shows the 25 earthquake ground motions considered in this study. Every earthquake contains two directions (E–W and N–S). As a consequence, the total number of records used is 50. As mentioned, there are two processes in the CNN method—the training and the validation processes. For this reason, the records are subdivided into two groups. The number of records for the training is 40 (20 earthquakes) and the number of records for the validation is 10 (5 earthquakes). This obeys the split ratio recommended for typical CNN procedures (80% training records and 20% validation records). The earthquakes are selected randomly to avoid extracting the same characteristics between different records. Figure 17 shows the acceleration response spectrum of the 50 records scaled to have the same values at the fundamental period of the structure (T_1_ = 0.5 s) as Sa (T_1_) = 100 gal.

#### 2.4.3. Scale Factor of Records

The linear and nonlinear behavior of the structure is obtained by using different intensities of earthquake ground motions. Thus, the records are scaled to include a wide range of earthquake intensity. In order to evaluate the range of the scale factors, an incremental dynamic analysis with the structural responses for damage identification is conducted by taking into account the variation of the Peak Ground Acceleration (PGA), and the ordinate of the response acceleration spectrum evaluated on the fundamental period of the structure (Sa(T_1_)).

**Figure 18:**
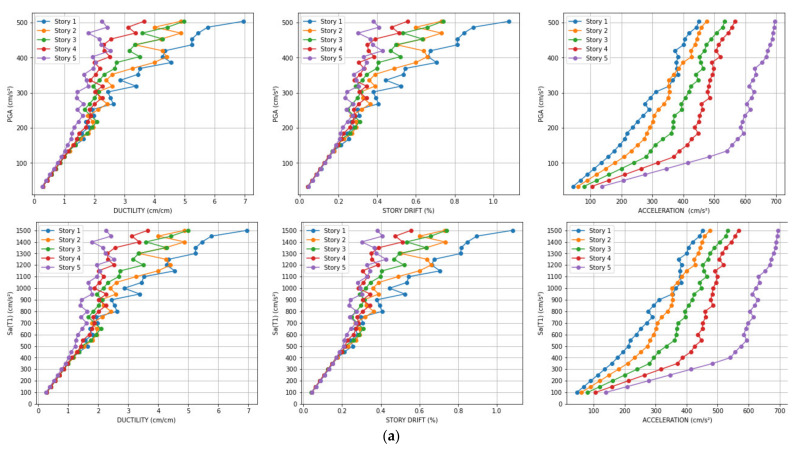

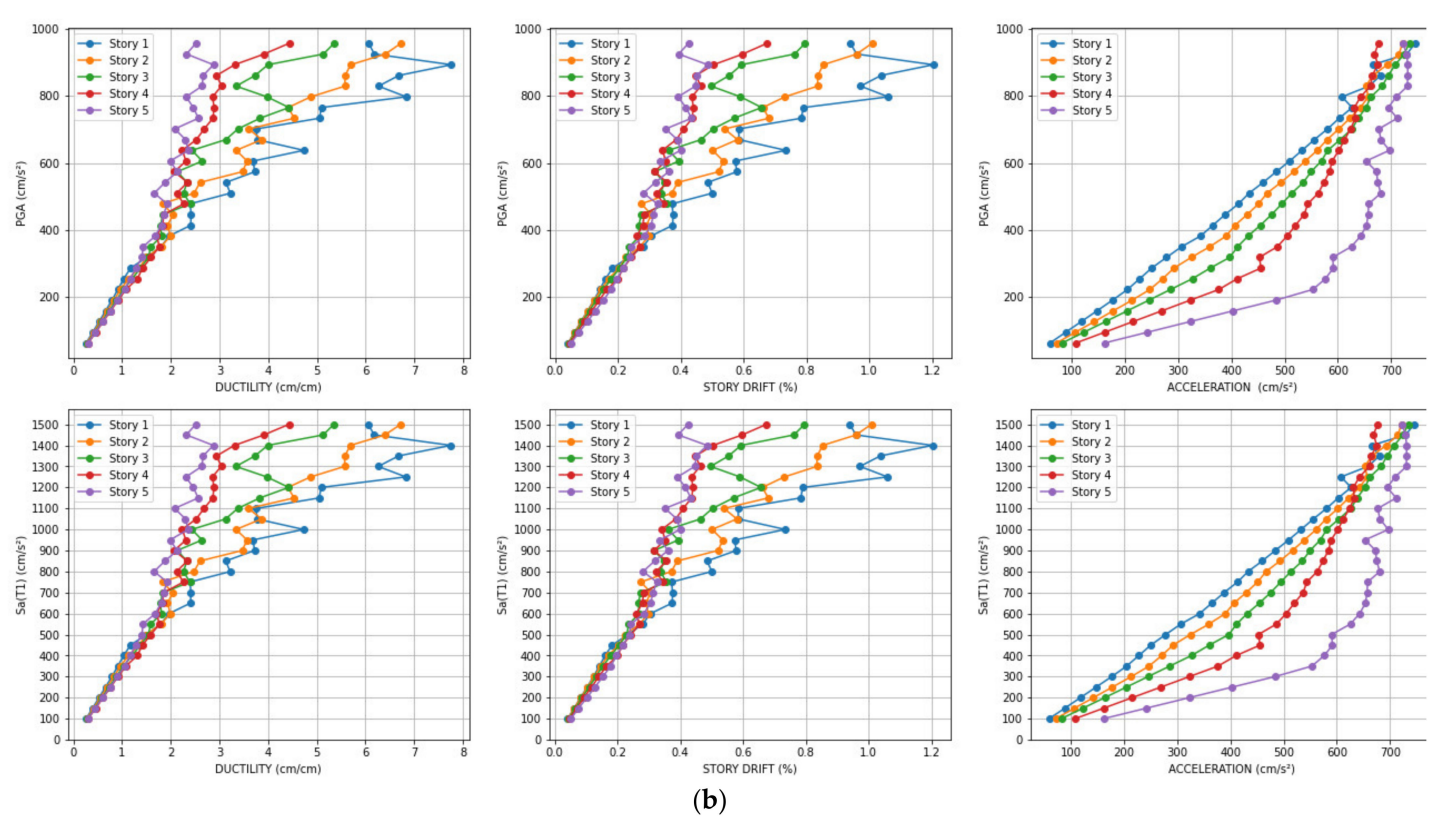
(**a**) Incremental structural responses for damage identification in each story with “El Centro 1940” input ground motion; (**b**) Incremental structural responses for damage identification in each story with “Northridge” input ground motion.

**Figure 19:**
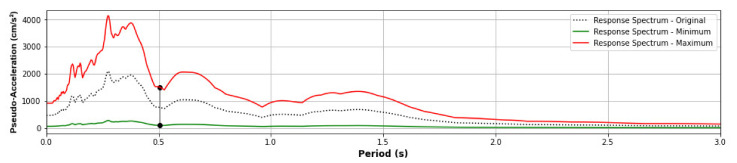
Acceleration response spectra of the “Loma Prieta” input ground motion. The red line is with the maximum scale factor such that it produces Sa(T_1_) = 1500 gal, the green line is with the minimum scale factor such that it produces Sa(T_1_) = 100 gal, and the black dashed line considers the original input ground motion.

Figure 18 presents the incremental structural responses for damage identification (ductility ratio, story drift ratio, and acceleration) in each story for the input ground motion “El Centro 1940” (Figure 18a) and “Northridge” (Figure 18b) using the same scale factor applied to Sa(T_1_) such that the minimum scale factor produces Sa(T_1_) = 100 gals and the maximum scale factor produces Sa(T_1_) = 1500 gals. Figure 18a shows the structural response under the maximum PGA of El Centro up to 500 gals. As shown in Figure 18b, the maximum PGA of Northridge must be around 1000 gals to achieve the same degree of response. Furthermore, the PGA of the threshold of the nonlinear behavior is around 150 gals in Figure 18a (El Centro) and 250 gals in Figure 18b (Northridge). On the other hand, the relationship between the responses and Sa(T_1_) is roughly the same in Figure 18a (El Centro 1940) and 18b (Northridge). Therefore, the Sa(T_1_) is more stable for characterizing the structural response of the structure. For this reason, the scale factor is based on Sa(T_1_) such that the minimum scale factor produces Sa(T_1_) = 100 gals, and the maximum scale factor produces Sa(T_1_) = 1500 gals and Sa(T_1_) = 1000 gals to train and validate the CNN model, respectively. Figure 19 shows the Acceleration Response Spectra of the “Loma Prieta” input ground motion considering the minimum and maximum scale factor and the original value.

### 2.5. Machine Learning Methodology

The methodology for predicting the structural responses for damage identification was as follows:

- The wavelet spectrum was obtained from the time–history acceleration response on the upper floor of the building. The frequency range was from 0.1/T_1_ to 5/T_1_, where T_1_ is the fundamental period of the case study structure (T_1_ = 0.5s), which is from 0.2 Hz to 10 Hz. This covered the high and low frequencies produced during high mode vibrations and nonlinear frequencies.
- There were two sets of scale factors for the training and validation of CNN processes.
- The training scale factor set was the minimum scale factor, which produces Sa(T_1_) = 100 gal, to the maximum, which produces Sa(T_1_) = 1500 gal, at increments of 50 gal.
- The validation scale factor set was the minimum scale factor, which produces Sa(T_1_) = 100 gal, to the maximum, which produces Sa(T_1_) = 1000 gal, at increments of 25 gal.
- There were 1160 structural analyses conducted for the training process by considering 40 records with 29 scale factors, while there were 370 structural analyses conducted for the validation process by considering 10 new records with 37 scale factors. Therefore, 1530 structural analyses carried out were used in this study.

The application of the methodology to predict the structural responses for damage identification was conducted as follows:

**TRAINING PROCESS**

- **STEP 01:** 40 training records are scaled with 29 scale factors per record. As a result, 1160 scaled records are generated.
- **STEP 02:** 1160 structural analyses are carried out for the structural model of the case study. As a result, 1160 absolute acceleration data on the upper floor are obtained. Additionally, the responses for damage identification (ductility ratio, story drift, and acceleration) are computed from the structural analyses for validating and calibrating the CNN model.
- **STEP 03:** 1160 wavelet spectra are obtained from the absolute acceleration of the previous step. The wavelet spectra are the input data for training the CNN model.
- **STEP 04:** The CNN model is trained for each structural response for damage identification (ductility ratio, story drift ratio, and acceleration).

**VALIDATION PROCESS**

- **STEP 01:** 10 validation records are scaled with 37 scale factors per record. As a result, 570 scaled records are generated.
- **STEP 02:** 370 structural analyses are carried out for the structural model of the case study. As a result, 370 absolute acceleration data on the upper floor are obtained. Additionally, the responses for damage identification (ductility ratio, story drift, and acceleration) are computed as reference outputs to validate the prediction.
- **STEP 03:** 370 wavelet spectra are obtained from the absolute acceleration of the previous step. The wavelet spectra are the input data for predicting the structural response for the damage identification using the trained CNN model.
- **STEP 04:** Prediction outputs are calculated using the CNN model for each structural response for damage identification (ductility ratio, story drift ratio, and acceleration).
- **STEP 05:** The reference and prediction outputs are compared.

## 3. Prediction and Validation of the Case Study

**Figure 20:**
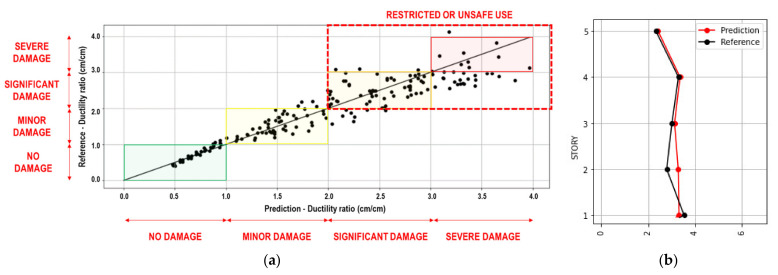
Example of the ductility ratio results (Petrolia California E–W record); (**a**) Comparison between prediction and reference values (points) and damage condition regions; (**b**) Prediction and reference ductility ratio of each story for a scale factor that produces Sa(T_1_) = 900 gal.

An example of the analysis results is shown in Figure 20. Figure 20a shows the ductility ratio results under the scaled Petrolia California E–W records, comparing the prediction (horizontal axis) and the reference (vertical axis). In the figure, the straight line represents the perfect prediction. The points represent the results of each story and scale factor defined in Section 2.1 and Section 2.4. Additionally, Figure 20a shows the regions that define the damage condition. The green, yellow, orange, and red regions represent the no damage, minor damage, significant damage, and severe damage conditions, respectively. The collapse condition is considered for any value greater than the severe damage condition. The dashed red rectangle encloses the region for any value that is greater than the minor damage condition and means that the use of the building is restricted or unsafe (condition for evacuating the building). Figure 20b shows an example of the prediction and reference values of each story for a scale factor that produces Sa(T_1_) = 900 gal.

**Figure 21:**
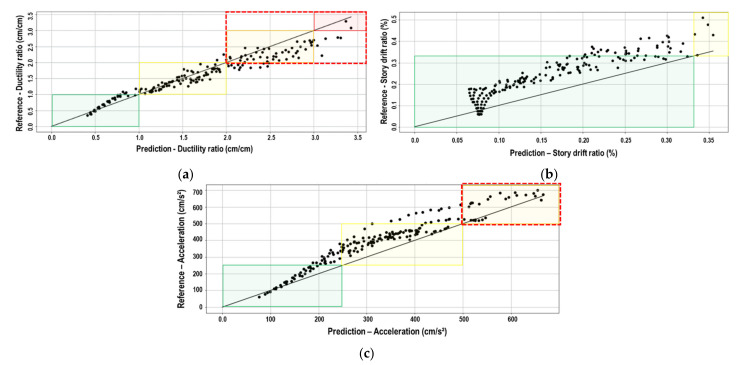
Comparison between reference and prediction of the Petrolia California N–S record for the validation process of (**a**) Ductility ratio; (**b**) Story drift ratio, and (**c**) Acceleration.

**Figure 22:**
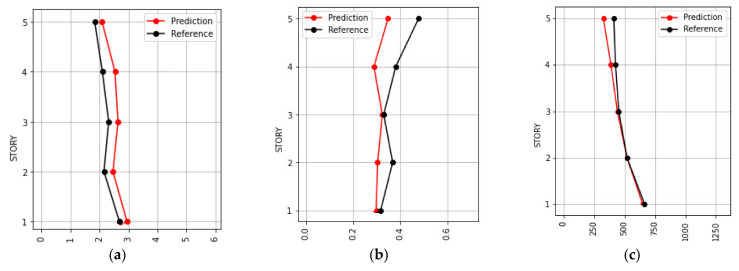
Prediction and reference values on each floor of the Petrolia California N–S record and scale factor that produces Sa(T_1_) = 875 gal for the validation process of (**a**) Ductility ratio; (**b**) Story drift ratio, and (**c**) Acceleration.

Figure 21 shows the results of the ductility ratio, story drift ratio, and acceleration for the validation process under the scaled Petrolia California N–S records. The regions that define the damage condition are also shown in the figure. As seen in Figure 21b, the story drift ratios do not reach the significant damage, severe damage, and collapse condition. Likewise, the restricted or unsafe use condition is not reached. Figure 22 shows the prediction and reference values of the ductility ratio, story drift ratio, and acceleration on each floor considered under the same record for a scale factor that produces Sa(T_1_) = 875 gal.

The coefficient of correlation (*r*) is used to measure the accuracy of the CNN model in this study, and it is defined as shown in Equation (3):

(3) $$r=\frac{\frac{1}{N}\cdot \sum_{i}^{N}\left(y_{pred,\;i}-\bar{y_{pred}}\right)\left(y_{ref,\;i}-\bar{y_{ref}}\right)}{\sqrt{\frac{1}{N}\cdot \sum_{i}^{N}{\left(y_{pred,\;i}-\bar{y_{pred}}\right)}^{2}}\cdot \sqrt{\frac{1}{N}\cdot \sum_{i}^{N}{\left(y_{ref,\;i}-\bar{y_{ref}}\right)}^{2}}}$$

where *y_pred_* is the prediction output by the CNN model, *y_ref_* is the reference output by the structural analysis, $\bar{y_{pred}}$ and $\bar{y_{ref}}$ are the mean of *y_pred_* and *y_ref_*, respectively, and *N* is the number of samples. Table 6 shows the *r*-values for the validation process. The average values of the *r*-values of the ductility ratio, story drift ratio, and acceleration are 0.905, 0.846, and 0.829, respectively. In particular, the accuracy of the estimation of the ductility ratio is the highest.

Two new ratios are introduced, the damage condition ratio (DCR) and the restricted or unsafe use ratio (RUUR), to examine the accuracy of the prediction of structural damage. The damage condition ratio (DCR) is defined as the ratio of the number of the predicted values and the number of reference values inside the damage condition region as shown in Equation (4). Likewise, the restricted or unsafe use ratio (RUUR) is defined as the number of the predicted values and the number of reference values inside the restricted or unsafe region as shown in Equation (5).

(4) $$\mathrm{DCR}\;=\frac{\mathrm{No}.\;\mathrm{of}\;\mathrm{Predicted}\;\mathrm{values}\;\mathrm{inside}\;\mathrm{the}\;\mathrm{damage}\;\mathrm{condition}\;\mathrm{region}}{\mathrm{No}.\;\mathrm{of}\;\mathrm{Reference}\;\mathrm{values}\;\mathrm{inside}\;\mathrm{the}\;\mathrm{damage}\;\mathrm{condition}\;\mathrm{region}}\cdot 100\%$$

(5) $$\mathrm{RUUR}=\frac{\mathrm{No}.\;\mathrm{of}\;\mathrm{Predicted}\;\mathrm{values}\;\mathrm{inside}\;\mathrm{the}\;\mathrm{restricted}\;\mathrm{or}\;\mathrm{unsafe}\;\mathrm{use}\;\mathrm{region}}{\mathrm{No}.\;\mathrm{of}\;\mathrm{Reference}\;\mathrm{values}\;\mathrm{inside}\;\mathrm{the}\;\mathrm{restricted}\;\mathrm{or}\;\mathrm{unsafe}\;\mathrm{use}\;\mathrm{region}}\cdot 100\%$$

**Figure 23:**
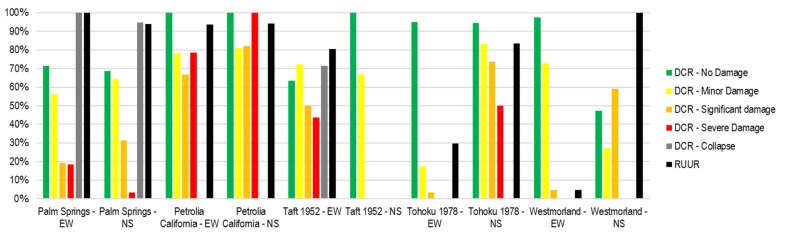
DCR and RUUR for ductility ratio of the validation process.

Figure 23 shows the comparison of the DCR and RUUR for the ductility ratio. In general, the DCR of no damage and collapse condition are larger and more accurate than others. In most cases, RUUR has high precision—greater than 80%. Notice that DCR and (or) RUUR for some records is not reached because the structural response is not over the limit for being measured.

**Figure 24:**
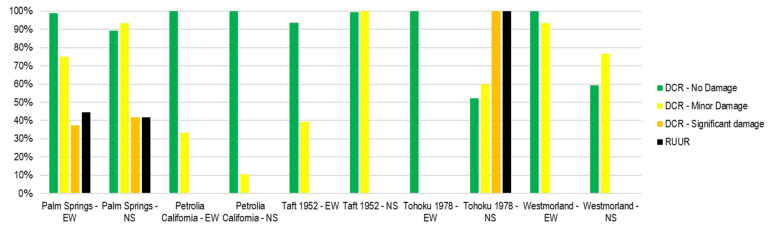
DCR and RUUR for story drift ratio of the validation process.

Figure 24 shows the comparison of the DCR and RUUR for the story drift ratio. In general, the DCR of no damage and minor damage condition are larger and more accurate than other conditions. Few data reach DCR of severe damage and collapse conditions.

**Figure 25:**
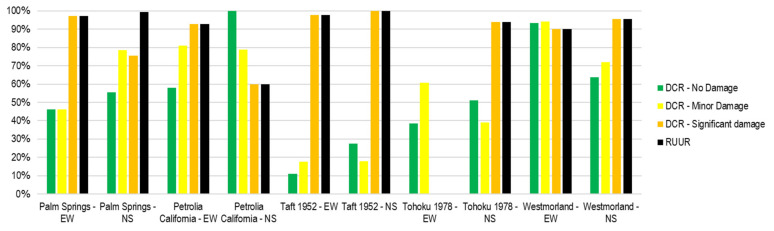
DCR and RUUR for the acceleration of the validation process.

Figure 25 shows the comparison of the DCR and RUUR for the acceleration. In general, the DCR of significant damage condition is larger and more accurate than others. Few data reach DCR of severe damage and collapse conditions. In most cases, RUUR has high precision—greater than 90%.

## 4. Conclusions and Discussion

In this study, a method is proposed with which to estimate the damage of a building by applying a machine learning method from the acceleration response at the upper floor of the building. The results of this research are summarized as follows:

- The maximum ductility factor, inter-story drift ratio, and maximum response acceleration of each floor were predicted via a CNN model using the acceleration record at the upper floor of the building.
- The wavelet spectrum of the acceleration record of the upper floor of the building was used as the input of the CNN model to account for the non-stationarity of both the amplitude and frequency of the building response.
- A CNN model was trained for the linear to nonlinear response of a building by inputting two horizontal components of 20 different earthquake ground motions with varying scales. The trained CNN model was then validated by inputting the two-directional horizontal components of five different earthquake motions to the building with different scales.
- The correlation coefficients between the predicted values and the reference values by the CNN model exceeded 0.8 for all response values, confirming the high accuracy of the model.
- The damage information evaluated by the CNN model was classified according to the target performance of the building as “no damage”, “minor damage”, “significant damage”, and “severe damage”. Furthermore, new ratios, DCR and RUUR, are proposed to examine the accuracy of the prediction of structural damage.

Using this method, it is possible to estimate the degree of damage to a building immediately after an earthquake using only the record of accelerometers installed on the upper floor of the building. The results will be useful for countermeasures after an earthquake, such as evacuation and decisions on the continued use of the building.

## Figures and Tables

**Table 1:** Proposal of damage condition according to the structural response for damage identification.

Damage Condition	No Damage	Minor Damage	Significant Damage	Severe Damage	Collapse
Ductility ratio	<1	≥1.0 but <2.0	≥2.0 but <3.0	≥3.0 but <4.0	≥4.0
Story drift ratio	<1/300	≥1/300 but <1/150	≥1/150 but <1/100	≥1/100 but < 1/75	≥1/75
Acceleration (gal)	<250	≥250 but <500	≥500 but <1000	≥1000 but < 1500	≥1500

**Table 2:** CNN architecture for the structural response prediction method.

No. of Layer	No. Kernels	Kernel Size	Padding	Kernel Initializer	Activation Function
Convolutional Layer 01	10	10 × 10	Same	He Normal	ReLU
Convolutional Layer 02	8	3 × 3	Same	He Normal	ReLU
Convolutional Layer 03	8	3 × 3	Same	He Normal	ReLU
Convolutional Layer 04	8	3 × 3	Same	He Normal	ReLU
Convolutional Layer 05	8	3 × 3	Same	glorot_uniform	ReLU
Convolutional Layer 06	8	3 × 3	Same	glorot_uniform	ReLU
Convolutional Layer 07	8	3 × 3	Same	glorot_uniform	ReLU
Convolutional Layer 08	8	3 × 3	Same	glorot_uniform	ReLU
Convolutional Layer 09	8	3 × 3	Same	glorot_uniform	ReLU
Convolutional Layer 10	8	3 × 3	Same	glorot_uniform	ReLU
Convolutional Layer 11	8	3 × 3	Same	glorot_uniform	ReLU
Convolutional Layer 12	8	3 × 3	Same	glorot_uniform	ReLU
Convolutional Layer 13	8	3 × 3	Same	glorot_uniform	ReLU
Convolutional Layer 14	8	3 × 3	Same	glorot_uniform	ReLU
Convolutional Layer 15	8	3 × 3	Same	glorot_uniform	ReLU
Convolutional Layer 16	8	3 × 3	Same	glorot_uniform	ReLU
Convolutional Layer 17	8	3 × 3	Same	glorot_uniform	ReLU

**Table 3:** Structural configuration of the case study.

Description	Nomenclature (Units)	Value
Number of stories	N	5
Story height	h (m)	4
Building height	H (m) = h × N	20
Width	B (m)	30
Area of floor	A = B^2^ (m^2^)	900
Weight per floor area	w (kN/m^2^)	12
Weight of floor	W (kN)	1080
Fundamental Period	T_1_ (s)	0.5

**Table 4:** Parameters of the bilinear hysteresis model used in the case study.

Story	ki (kN/mm)	Qi (kN)
5	87	587.87
4	157	954.15
3	209	1240.66
2	244	1460.87
1	261	1620.00

**Table 5:** Earthquake ground motions.

No.	Name	Location	Station Reference	Magnitude	Date
	Training Process *				
01	Anza_01	USA	33.706N, 116.235W/Ground Floor: South Wing	Mw = 5.2	12/06/2005
02	Anza_02	USA	33.706N, 116.235W/Roof: Center Hallway of S. Wing	Mw = 5.2	12/06/2005
03	El Centro 1940	USA	Imperial Valley Earthquake	Mw = 6.9	18/05/1940
04	Kobe 1995	Japan	Great Hanshin Earthquake/Kobe Marine Observatory	Mw = 6.9	17/01/1995
05	Loma Prieta_01	USA	36.974N, 121.952W/Capitola—Fire Station	Ms = 7.1	18/10/1989
06	Loma Prieta_02	USA	36.973N, 121.572W/Gilroy #1—Gavilan College	Ms = 7.1	18/10/1989
07	Loma Prieta_03	USA	36.987N, 121.536W/Gilroy #3—Gilroy Sewage Plant	Ms = 7.1	18/10/1989
08	Loma Prieta_04	USA	37.046N, 121.803W/Corralitos—Eureka Canyon Rd.	Ms = 7.1	18/10/1989
09	Loma Prieta_05	USA	37.118N, 121.550W/Coyote Lake Dam	Ms = 7.1	18/10/1989
10	Loma Prieta_06	USA	37.255N, 122.031W/Saratoga—Aloha Ave.	Ms = 7.1	18/10/1989
11	Northridge_01	USA	34.068N, 118.439W/Los Angeles—UCLA Grounds	Mw = 6.7	17/01/1994
12	Northridge_02	USA	34.236N, 118.439W/Arleta—NordHoff Ave. Fire Station	Mw = 6.7	17/01/1994
13	Northridge_03	USA	34.387N, 118.530W/Newhall—LA County Fire Station	Mw = 6.7	17/01/1994
14	Petrolia_01	USA	40.325N, 124.287W/Petrolia	Mw = 7.0	25/04/1992
15	Petrolia_02	USA	40.503N, 124.100W/Rio Dell—101/Painter St. Overpass	Mw = 7.0	25/04/1992
16	Petrolia Aftershock_01	USA	40.325N, 124.287W/Petrolia/04/26/92, 07:41:40 UTC	Ms = 6.6	26/04/1992
17	Petrolia Aftershock_02	USA	40.325N, 124.287W/Petrolia/04/26/92, 11:18:25 UTC	Ms = 6.6	26/04/1992
18	Petrolia Aftershock_03	USA	40.026N, 124.069W/Shelter Cove—Airport	Ms = 6.6	26/04/1992
19	Whittier_01	USA	34.037N, 118.178W/Los Angeles—Obregon Park	Ml = 6.1	01/10/1987
20	Whittier_02	USA	34.160N, 118.534W/Tarzana—Cedar Hill Nursery	Ml = 6.1	01/10/1987
	**Validation Process** *				
21	Palm Springs	USA	33.962N, 116.509W/Desert Hot Springs	Ml = 6.1	08/07/1986
22	Petrolia California	USA	40.325N, 124.287W/Petrolia	Ml = 5.9	17/08/1991
23	Taft 1952	USA	Kern County, California Earthquake	Mw = 7.3	21/07/1952
24	Tohoku 1978	Japan	Miyagi Earthquake/Recorded at Tohoku University	Ms = 7.7	12/06/1978
25	Westmorland	USA	33.037N, 115.623W/Westmorland	Ml = 6.0	26/04/1981

* See Section 2.5 for details.

**Table 6:** Coefficient of correlation for the validation process.

No.	Record	Ductility Ratio	Story Drift Ratio	Acceleration
01	Palm Springs E–W	0.953	0.947	0.928
02	Palm Springs N–S	0.895	0.917	0.951
03	Petrolia California E–W	0.933	0.845	0.873
04	Petrolia California N–S	0.972	0.926	0.956
05	Taft 1952 E–W	0.872	0.771	0.417
06	Taft 1952 N–S	0.806	0.848	0.870
07	Tohoku 1978 E–W	0.833	0.466	0.562
08	Tohoku 1978 N–S	0.943	0.890	0.797
09	Westmorland E–W	0.925	0.969	0.985
10	Westmorland N–S	0.916	0.883	0.950
	Average	0.905	0.846	0.829

## Data Availability

The data presented in this study are available on request from the corresponding author.
